# Direct Targeting of Gene Regulators by Iridium(III) and Rhodium(III) Complexes

**DOI:** 10.1021/acs.accounts.5c00570

**Published:** 2025-10-28

**Authors:** Lei Wu, Lingtan Kong, Wanhe Wang, Chung-Hang Leung

**Affiliations:** † The State Key Laboratory of Mechanism and Quality of Chinese Medicine, Institute of Chinese Medical Sciences, 59193 University of Macau, Taipa, Macao 999078, China; ‡ Institute of Medical Research, 26487 Northwestern Polytechnical University, Xi’an, Shaanxi 710072, China; § Research & Development Institute of Northwestern Polytechnical University in Shenzhen, 45 South Gaoxin Road, Shenzhen 518057, China

## Abstract

Aberrant gene expression is frequently linked to the progression of various disorders and diseases, playing an instrumental role in pathological processes. Gene-regulation-related proteins, especially epigenetic enzymes and transcription factors, are critically involved in gene expression patterns. Therefore, targeting endogenous gene regulators presents novel approaches for potential therapeutic intervention.

Transition metal complexes have been extensively employed in diagnosis and treatment due to their distinctive properties. Organometallic iridium­(III) and rhodium­(III) complexes exhibit diverse structures, including photochemical and photophysical properties, kinetic stability, and the ability to interact specifically with biomolecules, particularly DNA and proteins, due to their selective steric engagement. Therefore, octahedral iridium­(III) and rhodium­(III) complexes represent attractive scaffolds for the design of probes and modulators of gene regulation.

Considering the complexity and spatiotemporal specificity of gene regulation, it is crucial to comprehend the interactions between target biomolecules, particularly protein–protein interactions (PPIs), to selectively modulate gene expression patterns. PPIs serve as hubs of cellular signaling flow during most biological activities, including gene expression processes. For example, regulators of histone modifications and transcription factors converge at transcription start sites (TSSs), where they engage unmodified substrates and assemble into transcriptional complexes. Discovering and regulating disease-related abnormal gene expression by modulating pivotal PPIs thus hold great promise. By leveraging their precisely defined steric scaffolds, organometallic iridium­(III) and rhodium­(III) complexes present a distinctive option for unveiling the biological roles of these proteins and identifying potential modulators.

In this Account, we discuss our recent work on discovering organometallic iridium­(III) and rhodium­(III) complexes for PPI-based gene modulation. First, we describe the interactions between these complexes and transcriptional-regulation-related proteins, including transcription factors and epigenetic enzymes, and discuss the key influences of the ligands and metal center on bioactivity. Second, we describe transition-metal-based conjugates that indirectly interact with gene regulators. Using the conjugation strategy, effective gene modulators can be developed without requiring extensive screening or compromising the ligand’s biological activity. Interestingly, modification of the iridium­(III) complex may transform the activity from agonistic to antagonistic, offering new insights into the development of gene regulation modulators. Additionally, these conjugates can serve as effective probes for screening gene regulation modulators with the use of time-resolved measurements to minimize interference from fluorescent molecules.

In summary, the studies discussed in this Account describe a series of organometallic iridium­(III) and rhodium­(III) complexes that specifically bind to gene regulatory proteins. These complexes act through precise three-dimensional binding instead of via redox modulation or covalent interactions. We expect that these complexes could provide the basis for the development of organometallic iridium­(III)- and rhodium­(III)-based drugs and advance our understanding of activity-based gene regulation.

## Graphical abstract

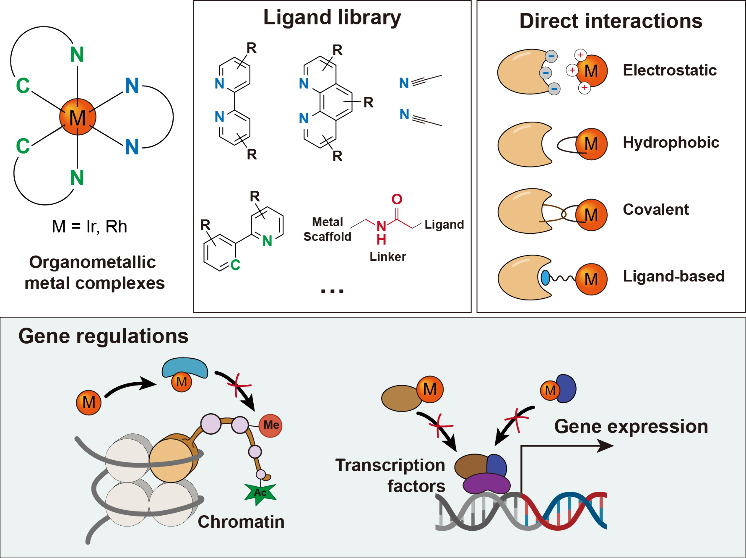

## Key References

-

Ma, D.-L. ; Liu, L.-J. ; Leung, K.-H. ; Chen, Y.-T. ; Zhong, H.-J. ; Chan, D. S.-H. ; Wang, H.-M. D. ; Leung, C.-H. Antagonizing STAT3 dimerization with a rhodium­(III) complex. Angew. Chem., Int. Ed. 2014, 53 (35), 9178–9182 10.1002/anie.20140468624889897.^1^ *This article describes the first example of a rhodium­(III) complex with two 4-(pyridin-2-yl)­benzaldehyde ligands and a 2,9-dimethyl-1,10-phenanthroline ligand for targeting the SH2 domain of STAT3 dimer, attenuating phosphorylation and dimerization and inhibiting signaling activity both in vitro and in vivo.*
-

Yang, G.-J. ; Wang, W. ; Mok, S. W. F. ; Wu, C. ; Law, B. Y. K. ; Miao, X.-M. ; Wu, K.-J. ; Zhong, H.-J. ; Wong, C.-Y. ; Wong, V. K. W. ; Ma, D. L. ; Leung, C.-H. Selective inhibition of lysine-specific demethylase 5A (KDM5A) using a rhodium­(III) complex for triple-negative breast cancer therapy. Angew. Chem., Int. Ed. 2018, 57 (40), 13091–13095 10.1002/anie.20180730529968419.^2^ *This article presents a novel rhodium­(III) complex-based inhibitor of KDM5A activity that modulates the interaction between H3K4me3 and KDM5A, emphasizing the importance of both ligands and the rhodium center in designing more active organometallic complexes.*
-

Li, G. ; Ko, C.-N. ; Li, D. ; Yang, C. ; Wang, W. ; Yang, G.-J. ; Di Primo, C. ; Wong, V. K. W. ; Xiang, Y. ; Lin, L. ; Ma, D.-L. ; Leung, C.-H. A small molecule HIF-1α stabilizer that accelerates diabetic wound healing. Nat. Commun.. 2021, 12 (1), 3363 34099651 10.1038/s41467-021-23448-7PMC8184911.^3^ *This work demonstrates a cyclometalated iridium­(III) metal complex as a stabilizer of hypoxia-inducible factor-1α via interrupting the VHL-HIF-1α interaction for wound healing. The complex bearing 2-phenylpyridine C^∧^N ligands and the dmeophen N^∧^N ligand possessed the highest activity at modulating HIF-1α transcriptional activity.*
-

Song, Y.-Q. ; Wu, K.-J. ; Zhang, Z. ; Liu, T.-M. ; Ko, C.-N. ; Zhu, W.-G. ; Ma, D.-L. ; Wang, W. ; Leung, C. H. Development of a sensitive luminescent probe to uncover new Bromodomain-containing protein 4 (BRD4) inhibitors in living cells. Chem. Eng. J. 2023, 463, 142356 .^4^ *This work demonstrates a new strategy using an iridium­(III) complex conjugated with a natural-product-like BRD4 inhibitor for activity-based drug screening, which only engages BRD4 within cells instead of other BET family members.*
-

Niu, D. ; Wu, X. ; Zhang, Y. ; Wang, X. ; Chan, D. S.-H. ; Jing, S. ; Wong, C.-Y. ; Wang, W. ; Leung, C.-H. Tailoring obeticholic acid activity by iridium­(III) complex conjugation to develop a farnesoid X receptor probe. J. Adv. Res. 2024, 71, 307–316 39490736 10.1016/j.jare.2024.10.028PMC12126729.^5^ *This article proposes an iridium­(III) complex–OCA conjugate that can reverse the activity of OCA from agonistic to antagonistic as well as prevent FXR–RXR heterodimerization while promoting the homodimerization of FXR in a metal-ion-dependent manner in living systems.*

## 1 Introduction

Gene expression in living organisms follows the instructions in genetic information. Gene products are often proteins produced by the processes of transcription and translation. Disruption of these biological processes leads to abnormal transcriptional programs, contributing to various diseases and disorders.^6,7^ Identification of the protein–protein interactions (PPIs) associated with disease traits therefore is thought to be critical in unveiling the mode of action and marking correlations between gene expression and disease-related traits.^8^ Various epigenetic regulators influence gene expression patterns via histone modifications, including acetylation, phosphorylation, and methylation.^8,9^ Transcription factors are recruited to specific genomic locations, where they interact with cofactors to assemble transcriptional initiation complexes and initiate gene transcription. This coordinated action ultimately shapes phenotypic traits and enables cellular responses to environmental cues.^10^ A number of organic small molecules, mostly nucleoside analogues or substrate derivatives, are currently used in therapeutic strategies targeting gene regulators. However, the limited tetrahedral, planar, or linear geometries of organic molecules resulting from the sp^3^, sp^2^, or sp hybridization of carbon atoms limits the structural architectures that can be constructed to engage the dynamic and steric interaction interfaces of biomacromolecules.^11^

Organometallic iridium­(III) and rhodium­(III) complexes exhibit significant advantages as potential therapeutic agents. The classical iridium­(III) and rhodium­(III) complexes consist of two cyclometalated ligands (C^∧^N) and one auxiliary ligand (N^∧^N).^11^ The cyclometalated ligands are commonly used to modulate the emission wavelength, while the auxiliary ligand serves to functionalize the complex.^12^ In addition, the synthesis of these complexes is often accomplished using a general synthetic method,^13^ which significantly reduces the synthetic complexity. Using a modular approach, different ligands can be easily introduced in order to optimize the structure of the complex for effective targeting of protein or enzyme active sites. Compared to platinum­(II) and ruthenium­(II) complexes, cyclometalated iridium­(III)/rhodium­(III) complexes possess several superior characteristics, such as easy synthesis, chemical stability, and octahedral coordination spheres.^14^ In addition, due to the populated metal-to-ligand charge-transfer transition (^3^MLCT) states, these complexes exhibit extended emission lifetimes, which can mitigate autofluorescence interference in bioimaging, and large Stokes shifts, which can avoid interference between excitation and emission light.^15^ Finally, the charge and lipophilicity characteristics associated with the metal core and auxiliary ligands endow them with high cell permeability and potential subcellular targetability.^16^ The above advantages form the basis for the excellent imaging and therapeutic capabilities of the iridium­(III) and rhodium­(III) complexes.

This Account focuses on advancements in discovering organometallic iridium­(III) and rhodium­(III) complexes for gene regulation through modulation of the PPIs of gene regulators (Figure 1). We also summarize strategies for designing organometallic complexes that modulate catalytic or interactive activities of target proteins through engineered coordination spheres, thereby altering their cellular distribution and regulatory functions. Importantly, we discuss a promising conjugation method that offers a new perspective for accelerating the development of metal-based modulators and PPI- or activity-dependent drug screening.

**Figure 1:**
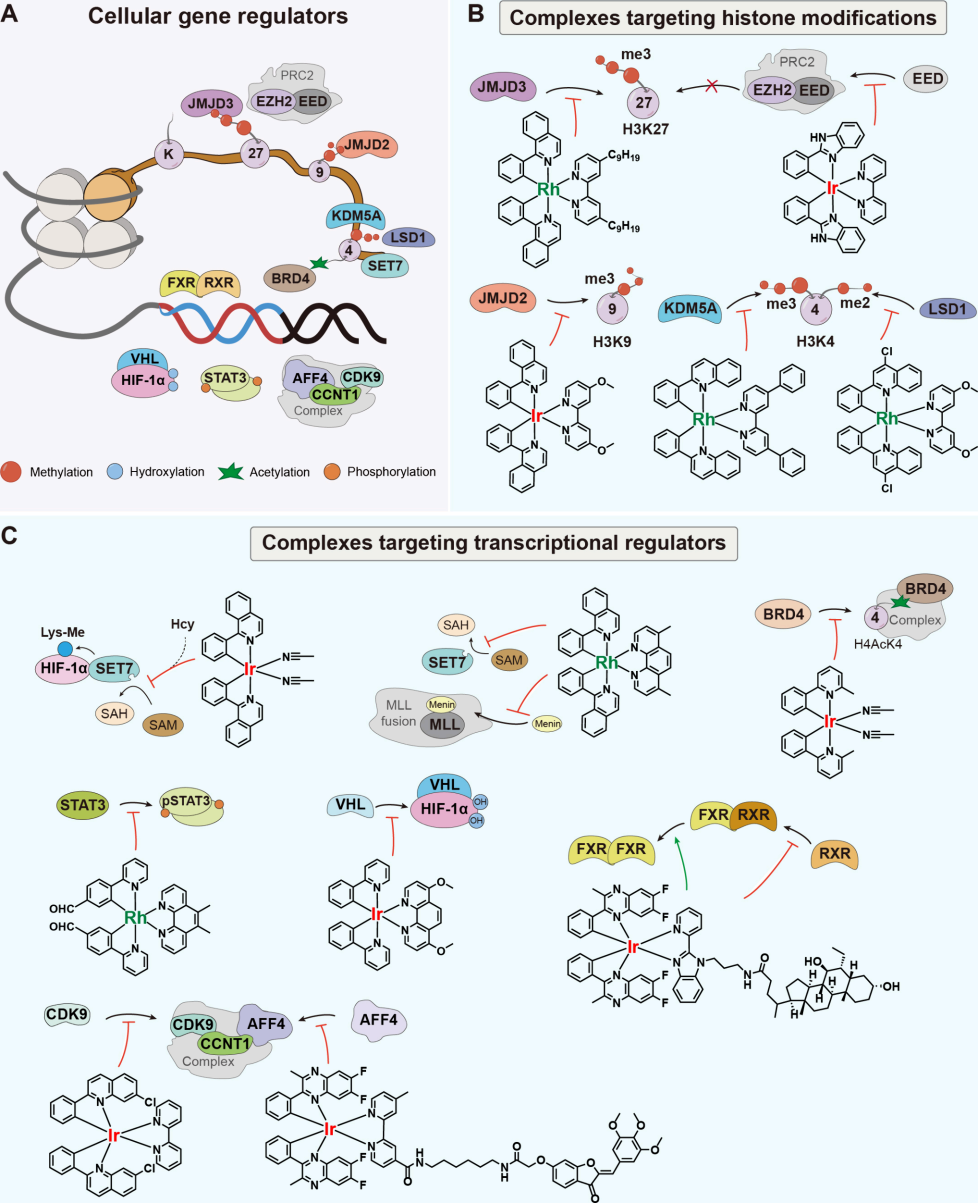
Schematic illustration of organometallic iridium­(III) and rhodium­(III) complexes for modulating gene regulators. (A) Overview of cellular gene regulators summarized in this Account. (B, C) Structures and mechanisms of iridium­(III) and rhodium­(III) complexes that target (B) histone modifications and (C) transcriptional regulators.

## 2 Rational Design of Organometallic Complexes with Precise Steric Features

Key milestones in the development of metal complexes as molecularly targeted agents include the 1952 discovery of a ruthenium­(II) complex that could effectively inhibit cholinesterase by Dwyer et al.^17^ In 1996, Jaouen et al. synthesized a novel ferrocene complex by loading the ferrocenyl group onto OH-tamoxifen, which can inhibit estrogen more effectively than tamoxifen.^18^ In the early 2010s, the Meggers group developed iridium­(III) and rhodium­(III) complexes based on staurosporine, in which the carbohydrate moiety is replaced by a metal complex fragment.^19,20^ The rigidity and spherical molecular architecture of octahedral metal complexes enabled them to fill protein pockets through shape and functional group complementarity, resulting in stronger inhibition of protein kinase compared with staurosporine alone. Concurrently, our group revealed that structurally unmodified iridium­(III) polypyridyl complexes could selectively inhibit tumor necrosis factor-alpha (TNF-α) via unique metal–ligand cooperativity.^21^ Based on the above advances, our group has been devoted to developing organometallic metal complexes through ligand optimization for targeting disease-related protein target interfaces.

In early research, we used known C^∧^N ligands and N^∧^N ligands to develop iridium­(III) and rhodium­(III) complexes that can inhibit specific proteins. We found that systematic structural optimization of ligands is required to tailor complexes to target proteins via target-specific binding. However, this process can require intensive and repetitive screening because the protein-binding activity of coordination complexes is significantly influenced by the electronic effects, steric constraints, and functional group modifications of their constituent ligands. To improve the efficiency of screening, we pioneered a novel strategy in which known small-molecule inhibitors were conjugated to N^∧^N ligands. This approach streamlined the process by requiring optimization of only the C^∧^N ligands and greatly improved the effectiveness and speed of screening. In addition, the biological activity of metal complexes is significantly influenced by alterations in the type of metal center due to their different sizes and charges.

### 2.1 Ligand Variability

At the beginning of our research into developing organometallic iridium­(III)/rhodium­(III) complex-based gene regulators, we employed a nonfocused screening method inspired by traditional high-throughput screening (HTS) drug discovery. Hence, a library of iridium­(III) and rhodium­(III) complexes with diverse structures was screened to ensure the discovery of potential hits, which was followed by further iterative optimization (Figure 2). Because of the typical kinetic inertness of their transition metal centers, organometallic iridium­(III)/rhodium­(III) complexes generally show ligand-dependent activity, whereas the metal center serves primarily to organize the organic ligands for the construction of sophisticated globular and rigid structures capable of recognizing proteins through precise molecular complementarity.^22^ In the initial work toward developing the first metal-based signal transducer and activator of transcription 3 (STAT3) inhibitor,^1^ we synthesized 11 rhodium­(III) and iridium­(III) complexes. Initial biological testing revealed that iridium­(III) complexes containing the C^∧^N ligand 4-(pyridin-2-yl)­benzaldehyde were the most promising candidates. In the second round of screening, a focused library of 26 derivatives was prepared for optimization studies. This demonstrated that having a C^∧^N ligand with aldehyde (CHO) substitution is key for targeting the Src Homology 2 (SH2) domain of STAT3, while N^∧^N coligands with larger π conjugation (e.g., dmphen, dmobpyphen) are favored, striking an optimal balance for cellular penetration (complex **1**). Further studies indicated that π conjugation (e.g., phenyl group, quinoline scaffold, phenanthroline scaffold, and benzimidazole scaffold) and electron-donating groups (e.g., methoxy groups) are favored for interaction between iridium­(III)/rhodium­(III) complexes and gene regulators, as demonstrated by iridium­(III) complex **3** based on 1-phenylisoquinoline paired with a 4,4′-dimethoxy-2,2′-bipyridine coligand for JmjC-domain-containing 2 (JMJD2) inhibition,^23^ rhodium­(III) complex **4** based on 4-chloro-2-phenylquinoline paired with a 4,4′-dimethoxy-2,2′-bipyridine coligand for lysine-specific demethylase 1 (LSD1) inhibition,^24^ rhodium­(III) complex **5** based on 2-phenylquinoline paired with a 4,4′-diphenyl-2,2′-bipyridine coligand for lysine-specific demethylase 5A (KDM5A) inhibition,^2^ rhodium­(III) complex **7** based on 2-phenylpyridine paired with a 4,7-dimethoxy-1,10-phenanthroline coligand for hypoxia-inducible factor-1α (HIF-1α) stabilization,^3^ and iridium­(III) complex **11** based on 2-phenylbenzimidazole paired with a 2,2-bipyridine coligand for embryonic ectoderm development (EED)-enhancer of zeste homologue 2 (EZH2) inhibition.^25^ Moreover, similar to small-molecule protein inhibitors,^26^ introduction of halogen groups can also raise the binding affinity of iridium­(III)/rhodium­(III) complexes, as seen in rhodium­(III) complex **4** for LSD1 inhibition and iridium­(III) complex **8** for cyclin-dependent kinase 9 (CDK9)–cyclin T1 (CCNT1) inhibition. Furthermore, alkyl groups may provide weak electron-donating interactions and steric interactions to enhance binding recognition between iridium­(III)/rhodium­(III) complexes and gene regulators, as observed in rhodium­(III) complex **6** based on 1-phenylisoquinoline paired with 4,4′-dinonyl-2,2′-bipyridine coligands for JMJD3 inhibition and rhodium­(III) complex **10** based on 1-phenylisoquinoline paired with 5,6-dimethyl-1,10-phenanthroline coligands for SET7/mixed-lineage leukemia protein (MLL) inhibition. Interestingly, iridium­(III) complexes with labile acetonitrile N^∧^N ligands show a distinct binding preference for gene regulators based on different C^∧^N ligands. Complex **2** based on 2-phenyl-6-methylpyridine favored bromodomain-containing protein 4 (BRD4) inhibition,^27^ while iridium­(III) complex **9** based on 1-phenylisoquinoline is selective for SET7/9 inhibition.^28^ Therefore, the ligands of these metal complexes necessitate the balance of steric bulk, hydrophobicity, and electronic properties, creating concise 3D architectures that are critical for selective protein inhibition through optimal binding affinity and functional activity.

**Figure 2:**
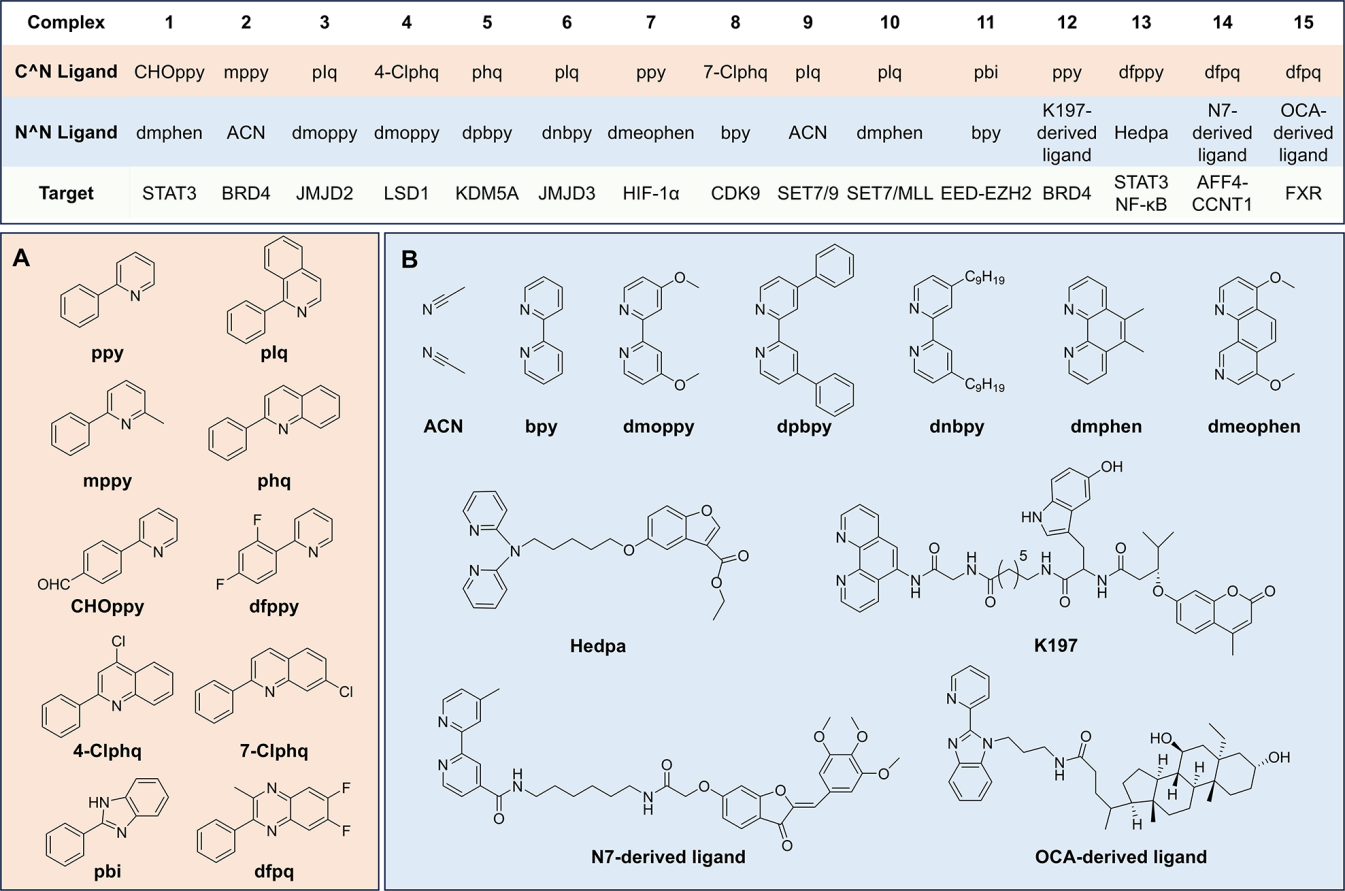
(top) Schematic of ligand variability and iridium­(III)/rhodium­(III) complexes **1**–**15**, with C^∧^N ligand, N^∧^N ligand, and molecular target indicated. (A) Chemical structures of C^∧^N ligands. (B) Chemical structures of N^∧^N ligands.

However, random screening for the discovery of metal-based gene regulators is labor-intensive and costly. Inspired by antibody–drug conjugate (ADC) and proteolysis-targeting chimera (PROTAC) methodologies, we conjugated bioactive molecules to an iridium­(III)/rhodium­(III) complex (Figure 2), which was facilitated by the availability of binders for some gene regulators, such as BRD4 (complex **12**).^4^ For targets without known inhibitors or modulators, we conducted virtual screening campaigns to obtain hits for metal conjugation. In the conjugation strategy, the linker length and C^∧^N ligands are key for their activity, while the N^∧^N ligand mainly serves as a site for linker modification. For example, in the case of STAT3 and NF-κB, iridium­(III) complex **13** with the C^∧^N ligand 2-(2,4-difluorophenyl)­pyridine containing fluorine atoms has stronger inhibitory activity.^29^ This is also observed in the case of AFF4–CCNT1, where iridium­(III) complex **14** with the C^∧^N ligand 6,7-difluoro-2-methyl-3-phenylquinoxaline containing fluorine atoms had optimal performance.^30^

### 2.2 Role of the Metal Center

The iridium­(III)/rhodium­(III) ion provides a central nexus to coordinate with the C^∧^N ligands and N^∧^N ligand to form precise three-dimensional architectures. In some cases, variation of the metal center significantly affects the biological activity of the complex. Organometallic iridium­(III) and rhodium­(III) complexes exhibit high stability in biological systems, allowing them to remain structurally intact and interact with gene regulators. Furthermore, these complexes demonstrate redox stability and can therefore modulate the activity of gene regulators independently of redox processes. These observations suggest that the difference in atomic size between iridium and rhodium can determine their biological activities toward proteins. This view aligns with the findings by Meggers that the metal center serves primarily to organize the organic ligands in three-dimensional space, generating structures with unique and defined shapes capable of recognizing proteins through precise molecular complementarity.^31^ Therefore, the metal center should be carefully investigated to achieve optimal activity (Figure 3).

**Figure 3:**
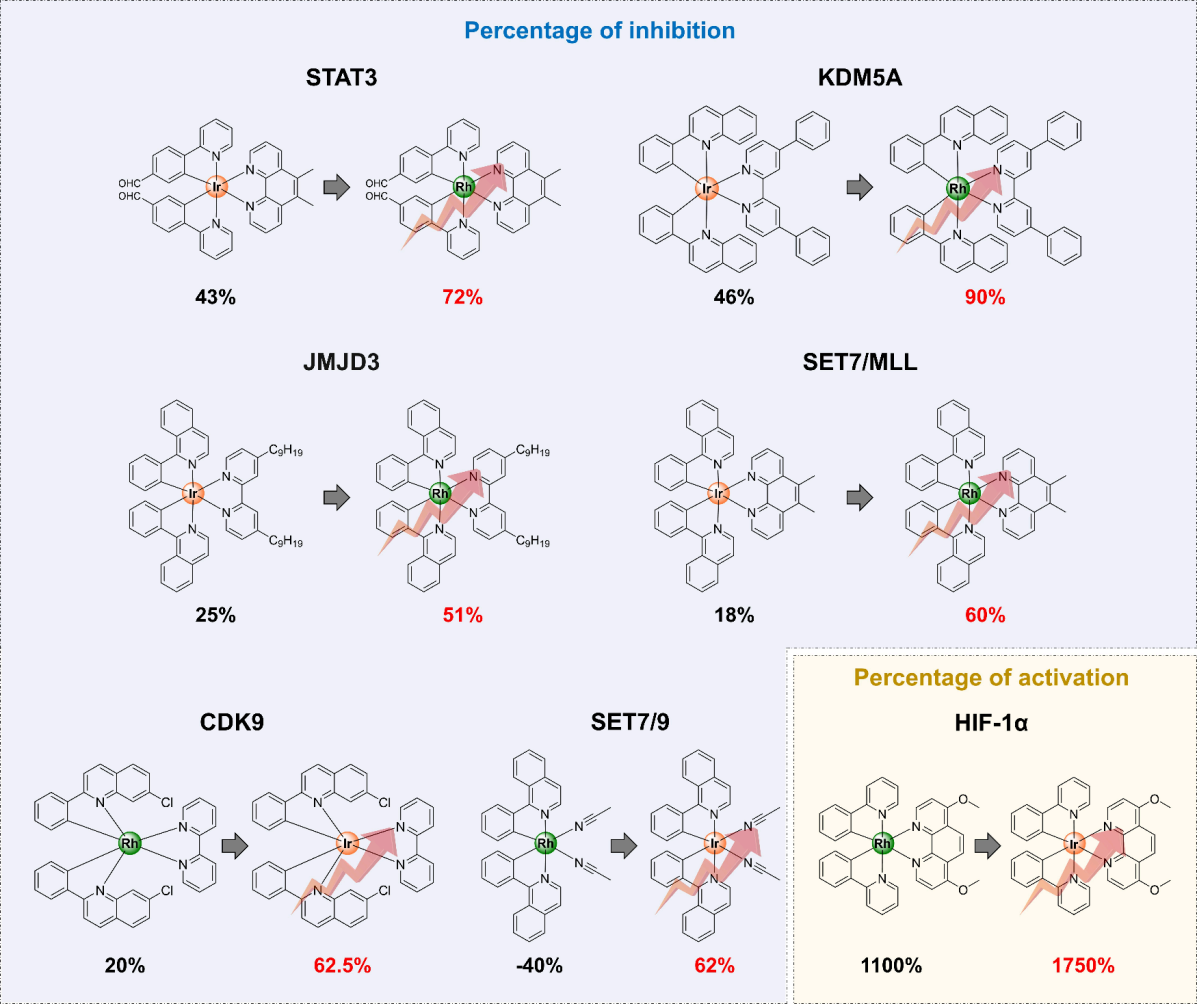
Effect of metal center identity (iridium­(III) vs rhodium­(III)) on the bioactivity of organometallic complexes toward gene regulators, including STAT3, KDM5A, JMJD3, SET7/9, and CDK9 (based on binding activity) as well as HIF-1α (based on promoter activity). The upper and lower panels show cases where the rhodium­(III) or iridium­(III) congener is superior, respectively.

Based on our recent work, we also discovered that changing the metal could even modulate the nature of the activity of transition metal complexes through cellular distribution differences. For example, the obeticholic acid-conjugated iridium­(III) complex **15** exhibits antagonistic activity, reversing the agonistic activity of obeticholic acid, while its ruthenium­(II) complex counterpart retains agonistic activity.^5^ Further mechanistic studies demonstrated that the differences in activity primarily arise from the cellular distributions of iridium­(III) complexes and ruthenium­(II) complexes. Specifically, iridium­(III) complex **15** fails to enter the nucleus and accumulates farnesoid X receptor (FXR) in the cytoplasm, whereas the ruthenium­(II) complex is able to enter and activate FXR. The iridium­(III) complex achieved near-infrared imaging of FXR in live cells and inhibited FXR nuclear translocation and downstream bile acid metabolism gene expression.

## 3 Variable Interfaces Determine the Bioactivity of Gene Regulators

The process of gene regulation is orchestrated by specific spatiotemporal assemblies of gene regulators. Typically, gene regulators aggregate at gene regulation hubs, where they identify and modify DNA sequences and interact with specific coactivators, mediators, or other transcription/epigenetic factors. The regulatory process is generally kick-started by an elaborate initiation machinery assembled near the TSSs.^32^ This machinery, comprising intricate components, is highly active and dynamically influences the flow of genetic information. The interaction interfaces between each component determine the bioactivity of gene regulators and facilitate diverse gene expression patterns via post-translational modifications (e.g., phosphorylation, acetylation, ubiquitination) and interactions (oligomerization, stereotypic exposure).^33,34^ Therefore, targeting the variable interfaces between these units can ultimately affect gene regulation and hence intervene in disease progression.

### 3.1 Direct Engagement with Gene Regulators

Back in the 1970s, cisplatin was first discovered to inhibit *Escherichia coli* cell division by Rosenberg and co-workers, and its derivatives were subsequently confirmed to interact with DNA and developed as potent anticancer therapeutics.^35,36^ In 1984, the Barton group pioneered a tris­(phenanthroline)­ruthenium­(II) complex that intercalates mismatched DNA.^37^ Subsequently, several transition metal complexes, such as ruthenium, rhodium, and iridium complexes, were also discovered to modulate protein kinase activity.^17,38–40^ Meggers et al. pioneered the development of rigid organoiridium scaffolds inspired from staurosporine, a natural product.^41^ This octahedral pyridocarbazole metal complex could bind to the active site of a protein kinase, with affinities and selectivities controlled by precisely arranged ligands. Our group has also previously developed a cyclometalated iridium­(III) complex that targets the interface of the TNF-α trimer, subsequently directly disrupting the TNF-α/TNFR interaction and inhibiting NF-κB transcription activity.^21^ This study then inspired us to directly modulate the activity of gene regulators via octahedral metal complexes.

We subsequently identified a distinct iridium­(III) complex as the first iridium­(III) scaffold-based inhibitor targeting JMJD2 histone demethylase (Figure 4A). The JMJD2 family enzymes (such as JMJD2D) rely on Fe­(II)/2-oxoglutarate to catalyze the demethylation of H3K9me3 and are abnormally activated in cancer.^23^ Utilizing metal-chelating scaffolds, we identified iridium­(III) complex **3** featuring two 1-phenylisoquinoline C^∧^N ligands and a 4,7-dmobpy N^∧^N ligand as a potent JMJD2D demethylase inhibitor. In contrast, the analogue complex bearing smaller 2-phenylpyridine C^∧^N ligands rather than the larger 1-phenylisoquinoline ligands of complex **3** suggested that smaller C^∧^N ligands may not be as effective at disrupting the JMJD2D catalytic interface. Complex **3** selectively inhibited JMJD2D activity through the synergistic effect of its 1-phenylisoquinoline C^∧^N ligands and 4,4′-dimethoxybipyridine N^∧^N ligand and does not depend on an iron chelation mechanism. At the cellular level, this compound upregulated the H3K9me3 methylation level of the tumor suppressor gene *p21* promoter by inhibiting the interaction between JMJD2D and H3K9me3 and induced apoptosis in lung cancer A549 cells. Compared with traditional organic inhibitors (such as *N*-oxalylglycine), it showed significant selectivity for JMJD2D over JMJD3, JARID, and HDAC enzymes, highlighting the unique advantages of iridium­(III) complexes in epigenetic regulation.

**Figure 4:**
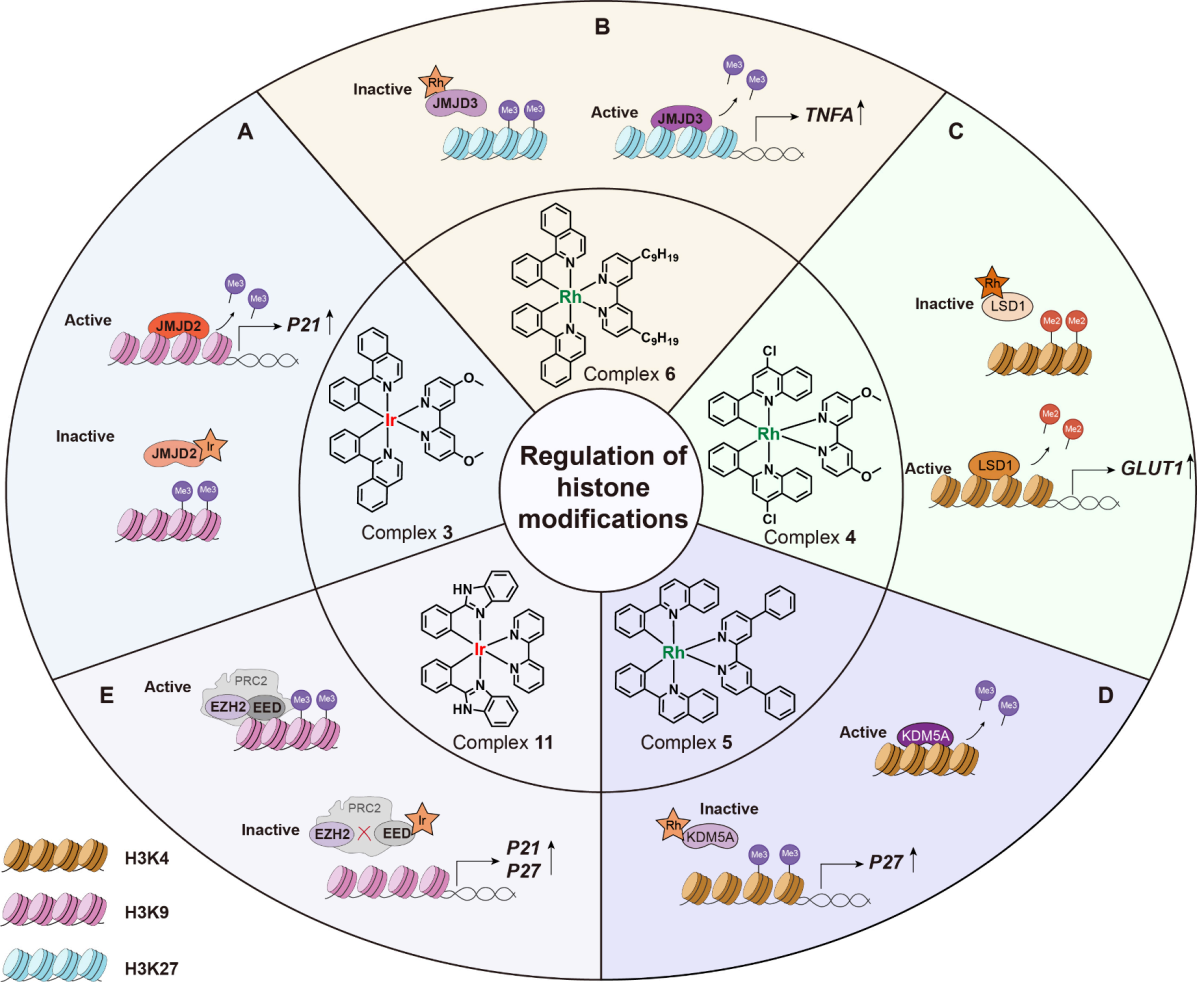
Octahedral iridium­(III)/rhodium­(III) complexes that directly target histone modification and regulate enzymatic activity. (A) Complex **3** targets JMJD2 and inhibits the demethylation of trimethylated H3K9. (B) Complex **6** targets JMJD3 and inhibits demethylation of trimethylated H3K27. (C) Complex **4** targets LSD1 and inhibits demethylation of dimethylated H3K4. (D) Complex **5** targets KDM5A and inhibits demethylation of trimethylated H3K4. (E) Complex **10** targets EED and disrupts the EED–EZH2 PPI, impeding the trimethylation of H3K9.

Unlike JMJD2D, which contributes to tumor formation, JMJD3 (KDM6B) is closely associated with inflammation. Activated JMJD3 erases trimethylation marks of H3K27me3, thus promoting the proinflammatory response. Mechanistically, the removal of H3K27me3 in the *TNFA* TTS region promotes the initiation of TNF transcription via enhancing the binding of RNA polymerase II.^42^ 2,2′-Bipyridine was reported as an excellent motif in compounds targeting DNA demethylases. Therefore, we evaluated a group of rhodium­(III) and iridium­(III) complexes based on this ligand and identified rhodium­(III) complex **6** bearing two 1-phenylisoquinoline C^∧^N ligands and a 4,4′-dinonyl-2,2′-bipyridine N^∧^N ligand as a selective JMJD3 inhibitor (Figure 4B).^43^ The extended 1-phenylisoquinoline C^∧^N ligands with a large aromatic interface and the hydrophobic N^∧^N ligand with long alkyl chains were critical for potency. Complex **6** exhibited an IC_50_ of 8.34 μM for JMJD3, outperforming its iridium­(III) congener complex (IC_50_ = 20.31 μM), possibly due to enhanced hydrophobicity and cellular uptake. Importantly, the isolated ligands showed no activity, emphasizing the necessity of a rhodium­(III)-coordinated scaffold. In LPS-stimulated macrophages, complex **6** selectively stabilized JMJD3, suppressed TNF-α production, and blocked JMJD3-H3K27me3 binding, as demonstrated via co-IP assays. Notably, complex **6** showed no cross-reactivity with KDM5A or JMJD2D, unlike the pan-inhibitor GSK-J4. This work established complex **6** as the first reported metal-based JMJD3 inhibitor, leveraging the ligand hydrophobicity and metal-center geometry for selective epigenetic modulation.

Lysine-specific demethylase 1 (LSD1) is a key epigenetic regulator that dimethylates H3K4 and is linked to prostate cancer.^44^ LSD1 was targeted by rhodium­(III) complex **4** featuring 4-chloro-2-phenylquinoline C^∧^N ligands and a 4,4′-dimethoxy-2,2′-bipyridine N^∧^N ligand (Figure 4C).^24^ Structural optimization revealed that chlorination of the C^∧^N ligand (complex **4**) enhanced LSD1 inhibition (over 10 times greater than that of the dechlorinated analogue complex), likely due to improved hydrophobic and electronic interactions with the catalytic pocket. Molecular docking suggested that complex **4** occupies a substrate-competitive site near Asp556, distinct from organic inhibitors like GSK2879552. In prostate cancer cells, complex **4** disrupted LSD1–H3K4me2 binding, elevated H3K4me2 levels at the *p21* and *FOXA2* promoters, and suppressed GLUT1 expression. Notably, complex **4** exhibited more than 100-fold selectivity over related enzymes (such as KDM2b, KDM7, and MAO), underscoring the role of ligand–metal coordination in achieving specificity. While complex **4** induced G0/G1 arrest in PC3 cells, it showed minimal toxicity in normal cells, positioning it as a pioneering metal-based LSD1 inhibitor with a tailored ligand for epigenetic therapy.

Whereas LSD1 mediates transcriptional silencing via H3K4me1/2 demethylation in corepressor assemblies, KDM5A targets H3K4me2/3 modifications, which are functional signatures of active promoters, to dynamically reshape transcriptional initiation, especially in triple-negative breast cancer (TNBC).^45^ TNBC poses considerable therapeutic difficulties due to its aggressive characteristics and the absence of targeted treatments. Epigenetic dysregulation, particularly aberrant histone methylation, is a hallmark of TNBC progression.^46^ KDM5A, which removes tri/dimethyl marks from histone H3K4 (H3K4me3/me2), is overexpressed in TNBC and drives tumorigenesis by silencing tumor suppressor genes.^47^ Rhodium­(III) complex **5** incorporating two 2-phenylquinoline C^∧^N ligands and a 4,4′-diphenyl-2,2′-bipyridine N^∧^N ligand was designed to antagonize KDM5A activity (Figure 4D).^2^ The 2-phenylquinoline C^∧^N ligands conferred superior inhibitory potency (IC_50_ = 23.2 nM) compared to analogous rhodium­(III) complexes bearing smaller 2-phenylpyridine ligands, likely due to enhanced steric complementarity and hydrophobic interactions with the catalytic interface of KDM5A. In contrast, the iridium­(III) congener complex with identical ligands exhibited reduced cellular uptake and activity, attributed to its lower hydrophobicity, as evidenced by shorter HPLC retention time. The rhodium­(III) center further facilitated cellular permeability and intracellular accumulation, driving selective inhibition of KDM5A over other demethylases (KDM1A, KDM4A, and KDM6B). In TNBC cells, complex **5** disrupted the KDM5A–H3K4me3 PPI, stabilized KDM5A, and elevated H3K4me3/me2 levels, leading to p27-mediated G1 arrest. Encouragingly, complex **5** dramatically reduced the size of the tumor in a xenograft model with minimal toxicity compared to cisplatin or doxorubicin. The p27 levels in groups treated with complex **5** were also increased, which is consistent with the *in vitro* results, reinforcing its potential as a novel metal-based KDM5A inhibitor with optimized ligand architecture for epigenetic targeting.

As another important epigenetic regulator in malignant tumors, polycomb repressive complex 2 (PRC2) reshapes chromatin by catalyzing the deposition of gene-repression-related H3K27me3 marks during various biological processes, including cell differentiation and embryonic development.^48^ EZH2, the central catalytic component of PRC2, trimethylates lysine 27 on H3 histone through its SET domain with the presence of EED, resulting in transcriptional silence of essential tumor suppressor genes and eventually promoting proliferation, metastasis, and invasion of tumor cells.^49,50^ We synthesized a series of organometallic iridium­(III) complexes comprising the benzimidazole moiety, a well-known bioactive structure of EED antagonists (Figure 4E).^25^ By varying the N^∧^N ligand, we found that the relatively small 2,2-bipyridine ligand was superior to larger ligands (e.g., 4,4′-diphenyl-2,2′-bipyridine and 4,7-dichlorophenanthroline). The results of optimizing the C^∧^N ligand also indicated that 2-phenylbenzimidazole was superior to the C^∧^N ligands 2-phenyl-4,5-dihydrooxazole, 1-phenyl-1*H*-pyrazole, and 2-phenylpyridine at EED–EZH2, which may be due to its more extensive hydrophobic cavity interacting with the EED–EZH2 interface. The most potent iridium­(III) complex (complex **11**) could selectively interact with EED and colocalize with EED in living cancer cells. Complex **11** decreased the accumulation of EZH2 at the promotor regions of *P21* and *P27*, thus promoting their expression. In the meantime, this iridium-based luminescent gene regulator also remodeled the EED–EZH2 nuclear translocation process and accumulated mRNA and protein levels of *P21* and *P27*, leading to significant antitumor activity *in vivo*.

In addition to histone demethylation, other modifications of histone, such as abnormal acetylation, are tightly correlated with tumor-related processes such as proliferation, apoptosis, and metastasis.^51,52^ BRD4 is an important member of the BET family that regulates the expression of oncogenes such as *c-MYC* by recognizing acetylated histones such as H4AcK4.^53^ We developed the iridium­(III) complex [Ir­(2-phenyl-6-methylpyridine)_2_(acetonitrile)_2_]^+^ (complex **2**) as the first irreversible metal-based BRD4 bromodomain inhibitor (Figure 5A).^27^ Through facile substitution of its acetonitrile ligand, the complex covalently binds to the histidine residue of the first bromodomain (BD1), interrupting its interaction with acetylated histone and inhibiting the expression of *c-MYC* and *Bcl-2*. Structural optimization showed that the 2-phenyl-6-methylpyridine C^∧^N ligand enhanced the binding through steric hindrance and hydrophobic interactions, while the iridium­(III) center was significantly more active than the rhodium­(III) analogue complex. In melanoma A375 and A2058 cells, the complex reduced chromatin binding of BRD4 in the *MYC* promoter and inhibited cell proliferation (IC_50_ to 12.5 μM and 3 μM, respectively). Complex **2** significantly decreased the expression of the *c-MYC* gene and extracellular-matrix-related genes and upregulated the genes in the VEGF signaling pathway in tumor tissues, which are all closely associated with BRD4 modulation, ultimately resulting in reduced tumor volume in mouse models. This study reveals innovative strategies for targeting epigenetic proteins through the covalent modifications of metal complexes.

**Figure 5:**
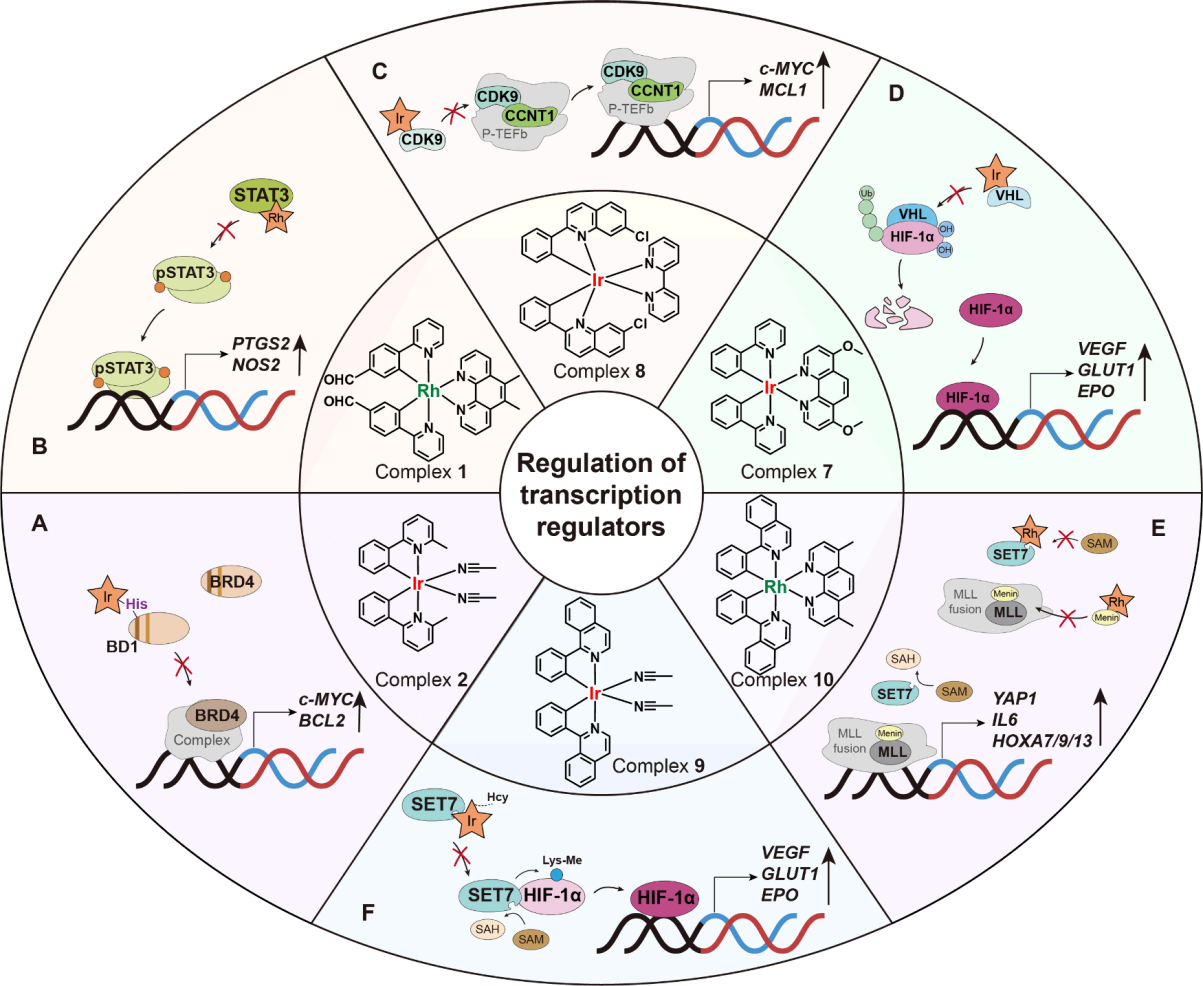
Octahedral iridium­(III)/rhodium­(III) complexes that target transcription regulators to modulate transcriptional activity. (A) Complex **2** covalently targets the BD1 domain of BRD4, inhibiting the formation of the BRD4 elongation complex. (B) Complex **1** targets the SH2 domain and hinders the dimerization of phosphorylated STAT3. (C) Complex **8** targets CDK9 and disrupts the CDK9–CCNT1 PPI in the P-TEFb complex. (D) Complex **7** targets VHL and rescues HIF-1α degradation by inhibiting VHL–HIF-1α engagement. (E) Dual targeting complex **10** interrupts the SET7–SAM and MLL–menin PPIs. (F) Complex **9** targets SET7 by generating an Hcy adduct that competitively disrupts the interaction between SET7 and SAM.

Compared to most traditional metal complexes that target nucleic acids, organometallic complexes engaging with gene regulators provide a novel approach to regulate abnormal gene expression.^33^ An early work focused on STAT3, which mediates numerous cellular responses, including interacting with tumor-associated regulators.^54^ To minimize nonspecific interaction with DNA, we developed polypyridyl-containing iridium and rhodium complexes since the substituted N^∧^N ligand has a relatively inert nature (Figure 5B). From a library of rhodium­(III) and iridium­(III) organometallic complexes bearing different polypyridyl-based ligands, we discovered that the rhodium center of complex **1** with 4-(2-pyridyl)­benzaldehyde as the C^∧^N ligand endows the cyclometalated complex with superior potency at inhibiting STAT3 binding to DNA compared to the congener complex with an iridium center. The lower cationic charge arising from the cyclometalated C^∧^N ligands likely reduced the DNA binding affinity, improving their selectivity for binding STAT3. Complex **1** inhibited STAT3 tyrosine-705 phosphorylation via targeting of the SH2 domain of STAT3, thus preventing interactions with phosphorylated Tyr residues and inhibiting further dimerization. Complex **1** dramatically reduced the phosphorylation of STAT3 in xenografted tumor tissues with negligible influences on JAK2 phosphorylation. The complex downregulated VEGF and exerted antiproliferative activities in tumor tissue through inhibiting STAT3-directed transcription and repressed angiogenesis in a mouse xenograft model.

CDK9, a core regulator of transcriptional elongation, forms a heterodimer with CCNT1 to phosphorylate the C-terminal domain of RNA Pol II, enabling the transcription of survival and proliferation genes such as *c-MYC* and *Mcl-1*. Dysregulation of the CDK9–CCNT1 complex drives pathological transcription in cancers, fueling tumor progression, metastasis, and therapy resistance.^55^ To target this interaction, the iridium­(III) complex **8** was designed with 7-chloro-2-phenylquinoline C^∧^N ligands and a 2,2′-bipyridine N^∧^N ligand, leveraging metal coordination to disrupt the CDK9–CCNT1 PPI (Figure 5C). Structural optimization revealed that the iridium­(III) center’s geometry and ligand architecture critically influenced binding: the chloro-substituted C^∧^N ligand provided steric and electronic complementarity to the hydrophobic interface of CDK9, while the planar, rigid N^∧^N ligand enhanced π stacking with Phe12 and van der Waals interactions with Gln71/Leu81. Substituting the C^∧^N ligand with bulkier analogues (for instance, 2-phenylquinoline) or modifying the N^∧^N ligand (with methyl or polar groups) reduced potency by up to 300-fold, emphasizing the importance of structural compatibility. Functional studies demonstrated that complex **8** disrupted CDK9–CCNT1 binding (*K*_d_ = 134 nM), suppressed *c-MYC* and *Mcl-1* transcription, and triggered apoptosis via caspase 3/9 activation. In metastatic TNBC models, complex **8** reduced the coprecipitation between CCNT1 and CDK9 and decreased mRNA levels of c-MYC and Mcl-1 in tumor tissues, which suggested that complex **8** disrupted the CDK9–CCNT1 PPI *in vivo*. Overall, tailoring C^∧^N/N^∧^N ligand combinations in metal complexes can achieve selective PPI inhibition, offering a paradigm for targeting transcriptionally abnormal cancers through noncatalytic kinase interfaces.

Diabetic wound healing remains a critical challenge due to impaired HIF-1α signaling, a key regulator of angiogenesis and tissue repair.^56^ The cyclometalated iridium­(III) complex **7** bearing two 2-phenylpyridine C^∧^N ligands and a 4,7-dimethoxy-1,10-phenanthroline N^∧^N ligand was identified as a potent stabilizer of HIF-1α via selective inhibition of the Von Hippel–Lindau (VHL)–HIF-1α PPI (Figure 5D).^3^ The iridium­(III) center and ligand architecture synergistically controlled the biological potency. Initial screening of rhodium­(III)/iridium­(III) complexes highlighted the importance of the brominated 2-(4-bromophenyl)­pyridine C^∧^N ligands and methoxy-substituted N^∧^N ligand for HIF-1α activation. Subsequent optimization demonstrated that replacing rhodium­(III) with iridium­(III) in complex **7** enhanced cellular uptake and activity (IC_50_ = 8.34 μM for JMJD3 inhibition), while substituting bulkier C^∧^N ligands (e.g., 2-phenylquinoline) or altering the N^∧^N substituents (e.g., removing methoxy groups) significantly reduced potency. The N^∧^N ligand in complex **7** conferred hydrophobicity and steric complementarity to engage the VHL binding pocket, as evidenced by thermal stabilization and a strong binding affinity for VHL (*K*_d_ = 1.08 μM). Notably, the isolated ligands (2-phenylpyridine and 4,7-dimethoxy-1,10-phenanthroline) showed no activity, confirming the necessity of the iridium­(III)-coordinated scaffold for target engagement. In diabetic mouse models, complex **7** stabilized HIF-1α, upregulated angiogenic factors (VEGF, GLUT1, and EPO), increased skin thickness, and accelerated wound closure by enhancing collagen deposition with minimal toxicity. This study highlights the strategic integration of iridium­(III) geometry and ligand hydrophobicity to develop metal-based PPI inhibitors, offering a novel therapeutic scaffold for diabetic wound healing via HIF-1α stabilization.

After the previous potent HIF-1α stabilizer was developed, we shifted our focus to the non-VHL degradation pathway. Studies have reported that the destabilization of HIF-1α is mediated through SET7-dependent lysine methylation, leading to the obstruction of diabetic wound healing. The SET7–*S*-adenosylmethionine (SAM) interaction drives HIF-1α degradation, disrupting transcriptional activation of pro-angiogenic genes, thereby highlighting the therapeutic potential of targeting this PPI to restore wound repair in diabetes.^56,57^ Therefore, our group developed iridium­(III) complex **9** comprising a redox-active iridium center coordinated with labile acetonitrile ligands and rigid 1-phenylisoquinoline moieties (Figure 5F) to enable dynamic ligand substitution with homocysteine (Hcy), a metabolite elevated in diabetes.^58^ This exchange generates a covalent complex **9**–Hcy adduct, which competitively disrupts the interaction between SET7 and its cofactor SAM, which is a critical step in the methyltransferase reaction. The iridium­(III) metal center not only stabilizes the three-dimensional arrangement of the ligands but also enhances the binding specificity to the SAM-binding pocket of SET7/9, showing high-affinity interactions (*K*_d_ = 1.06–1.54 μM). Specifically, the iridium­(III) core enhances hydrophobic and electrostatic complementarity with the SAM-binding site, while the 1-phenylisoquinoline ligand stabilizes π–π interactions with aromatic residues of Phe155, anchoring the complex within the catalytic domain. This dual ligand–metal synergy disrupts the methyltransferase activity of SET7, reducing HIF-1α K32 methylation and stabilizing HIF-1α under hyperglycemic hypoxia. Complex **9** upregulated VEGF, GLUT1, and EPO expression, accelerating angiogenesis and wound closure in diabetic models. Inspired by the unique properties of engagement between complex **9** and SET7, we combined the two rigid 1-phenylisoquinoline C^∧^N ligands of complex **9** with a 5,6-dimethyl-1,10-phenanthroline N^∧^N ligand from a bioavailable selective inhibitor of the MLL–menin PPI^59^ to obtain a dual targeting rhodium­(III) scaffold, complex **10** (Figure 5E).^60^ The hybrid complex **10** bound to the SAM binding pocket of SET7 and disrupted the menin–MLL PPI, eventually inhibiting the activity of the androgen receptor for treating castration-resistant prostate cancer. These designs indicate the strategic use of labile ligands and metal-directed scaffolds to target PPIs, offering a new paradigm for epigenetic metallodrugs in metabolic disorders.

### 3.2 Indirect Targeting of Gene Regulators through Complex-Based Conjugates

Over the past decade, our team has screened and developed various series of iridium/rhodium complexes for directly regulating disease-related gene expressions. From coordination between ligands and metals to interactions between complexes and protein interfaces, we have meticulously deciphered the structure–activity relationships (SARs) required for each separate target. However, optimizing these complexes remains a labor-intensive process, as we must synthesize and evaluate a wide range of ligands to accommodate the diverse structures and spatiotemporal distributions of gene regulators in cells. Therefore, in recent years, we dedicated ourselves to developing metal-complex-based conjugates consisting of three parts: metal complex scaffold, variable linker, and targeting unit. The metal complex can serve as both a distinct molecular probe and a functional inhibitor because of its unique photophysical properties and stereochemical configuration. When conjugated to a targeting unit, the metal complex can also influence the endocytosis pathway and subcellular distribution of the conjugate since metal complexes can often permeate cellular membranes. While the targeting unit facilitates specific binding to gene regulators, the potency and selectivity of the conjugate can be further modulated by tuning the linker’s properties, such as its length and polarity. Through this indirect targeting strategy, we aimed to achieve more precise and effective regulation of the gene regulator activity.

To demonstrate the feasibility of metal-based conjugates, we first developed a conjugate targeting the AFF4–CCTN1 PPI, a component within the super elongation complex (SEC).This PPI drives oncogenic transcription in TNBC by activating MYC, a key regulator of proliferation, metastasis, and cancer stem cell (CSC) phenotypes.^61^ Dysregulation of this PPI promotes tumor aggressiveness and resistance to conventional therapies, highlighting CCNT1 as a critical therapeutic target.^62^ However, the lack of selective inhibitors for this interaction has hindered clinical progress. To address this gap, we identified a novel scaffold from a natural product analogue library using a reported inhibitor as the docking basis, yielding N7, a potent AFF4–CCNT1 PPI inhibitor. N7 was then conjugated with an iridium­(III) scaffold to create complex **14**, a theranostic agent combining therapeutic and diagnostic functionalities (Figure 6A). The iridium­(III) core imparts near-infrared luminescence, enabling real-time imaging of CCNT1 in TNBC cells and tumor spheroids while distinguishing cancerous cells (with a high level of CCNT1 expression) from normal cells. Complex **14** features a cyclometalated iridium­(III) center coordinated with a 6,7-difluoro-2-methyl-3-phenylquinoxaline C^∧^N ligand and a flexible linker tethering the N7 moiety. The dfpq ligand’s extended aromatic system enhances π–π stacking with hydrophobic residues in the CCNT1 binding pocket, while the iridium­(III) center stabilizes the three-dimensional architecture. In contrast, the control complex, bearing a simpler 2-phenylpyridine C^∧^N ligand, exhibited negligible inhibitory activity, highlighting the critical role of the 6,7-difluoro-2-methyl-3-phenylquinoxaline ligand in achieving high-affinity binding (*K*_d_ = 0.35 μM) and selective CCNT1 engagement. The N7 moiety in complex **14** mimics the AFF4 LFAEP peptide, competitively occupying the CCNT1 binding site and disrupting the SEC-mediated *MYC* transcription. Chromatin immunoprecipitation (ChIP) assays validated this disruption, demonstrating reduced level of binding of AFF4 to the *MYC* promoter. Furthermore, complex **14** could suppress CSC biomarkers (CD44^+^/CD24^–^) and epithelial–mesenchymal transition (EMT) markers (e.g., N-cadherin) while upregulating E-cadherin in TNBC cells. In the TNBC allograft model, complex **14** colocalized with CCNT1 and disrupted the AFF4–CCNT1 PPI without significant change in their protein levels, leading to inhibited MYC expression. In addition, complex **14** impaired PCNA expression *in vivo*, which presumably accounts for suppressed tumor growth. These findings support the role of complex **14** as a promising scaffold for targeting SEC-driven malignancies and highlight the efficacy of metallodrugs at modulating PPIs through indirect transcriptional regulation.

**Figure 6:**
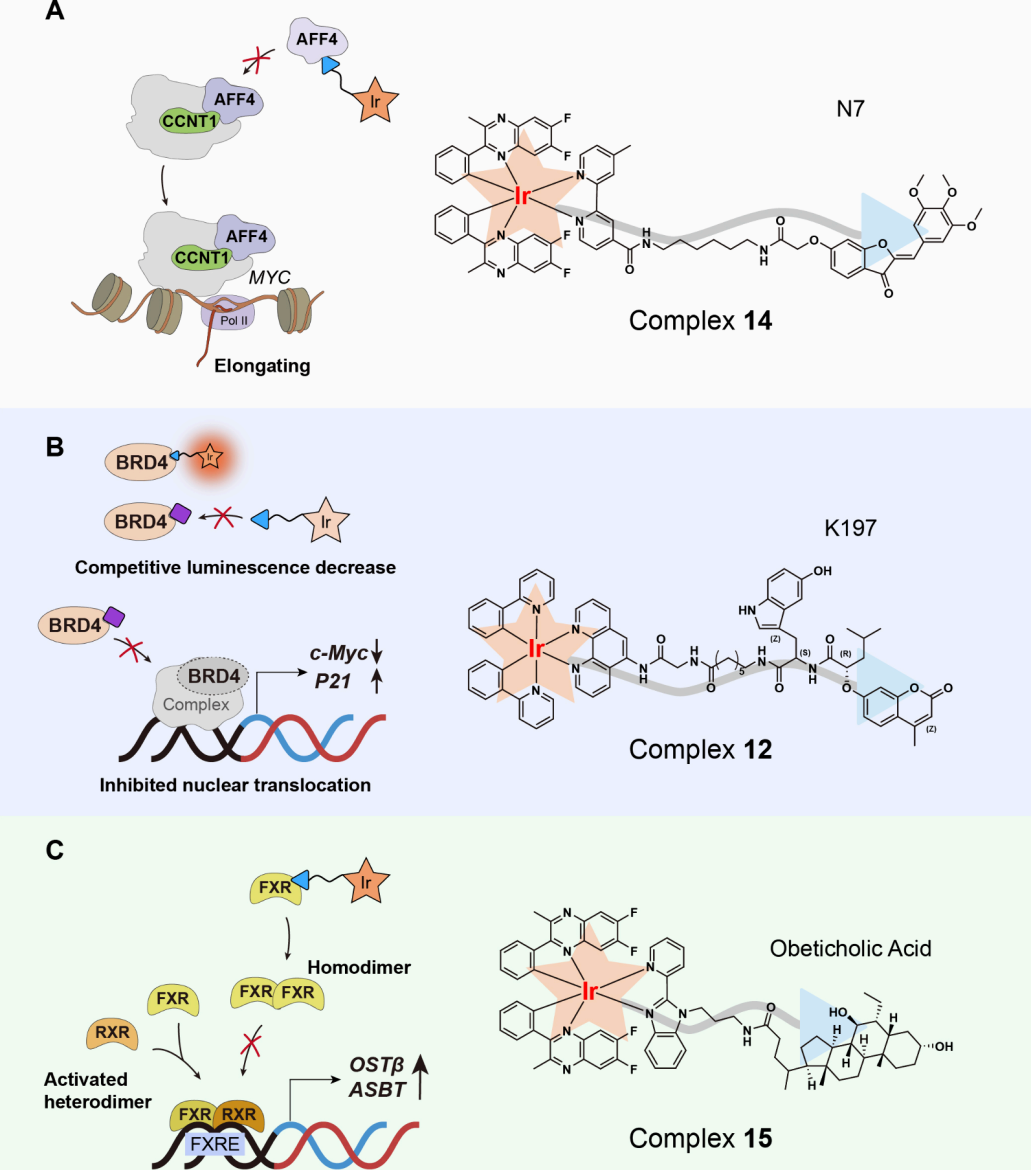
Iridium­(III) conjugates indirectly modulate transcription regulators via an active ligand. (A) Iridium­(III) conjugated N7 as an AFF4–CCNT1 PPI inhibitor disrupts oncogene elongation. (B) BRD4-targeting inhibitor conjugated with a cyclometalated iridium­(III) scaffold enables luminescence-based imaging and intracellular inhibitor discovery. (C) Obeticholic acid-based iridium­(III) conjugate targets FXR and inhibits its heterodimerization with RXR.

Melanoma, a highly aggressive and treatment-resistant form of skin cancer, remains a significant clinical challenge due to its propensity for metastasis and poor prognosis.^63^ BRD4 is a key epigenetic reader that drives oncogenesis by mediating PPIs with acetylated histones and transcription factors (for example, NF-κB and c-MYC), thereby regulating transcriptional elongation and cancer cell plasticity.^64^ Targeting BRD4’s PPIs offers a promising therapeutic strategy, but conventional fluorescence-based screening methods are hindered by autofluorescence interference and false positives. To address this, a luminescent iridium­(III) probe (complex **12**) was developed by conjugating a BRD4-targeting inhibitor (K197) to a cyclometalated iridium­(III) scaffold (Figure 6B). Complex **12** exhibited near-infrared emission with a long phosphorescence lifetime (τ = 862 ns) and wide Stokes shift, enabling time-resolved luminescence imaging to suppress background noise. CETSA and siRNA knockdown confirmed selective binding of complex **12** to BRD4 in melanoma A375 cells, with minimal cytotoxicity (IC_50_ > 50 μM). A proof-of-concept screen of 500 FDA-approved drugs identified azelastine, an H1 antagonist, as a BRD4 inhibitor. Azelastine disrupted BRD4 nuclear translocation, suppressed A375 cell viability, inhibited migration, and induced G0 and G1 cell-cycle arrest. Mechanistically, azelastine downregulated oncogenic targets (c-MYC, N-cadherin) and upregulated tumor suppressors (E-cadherin, p21), mirroring the effects of the canonical inhibitor JQ1. This study highlights complex **12** as a robust theranostic platform for real-time BRD4 imaging and inhibitor discovery, leveraging the photophysical advantages of iridium­(III) to overcome the limitations of fluorescence-based screens. The repurposing of azelastine further highlights the potential of metallodrugs in targeting PPIs for cancer therapy, offering a pathway to accelerate drug development for aggressive malignancies like melanoma.

In addition to dysregulation of tumor-associated gene expression, aberrant regulation also occurs in metabolic diseases. Dysregulated FXR signaling in intestinal epithelial cells exacerbates metabolic and inflammatory diseases,^65^ yet existing probes lack dual functionality for imaging and activity modulation. The cyclometalated iridium­(III) complex **15** was engineered by conjugating the FXR agonist obeticholic acid (OCA) to a luminescent iridium­(III) scaffold bearing 6,7-difluoro-3-methyl-2-phenylquinoline C^∧^N ligands and an alkyl linker (Figure 6C).^5^ SAR studies revealed that the iridium­(III) center and C^∧^N ligand architecture synergistically enabled near-infrared emission with robust photostability while reversing OCA’s agonism to antagonism. Complex **15** suppressed FXR nuclear translocation, disrupted FXR–RXR heterodimerization, and downregulated bile acid transporters (IBAP, OSTβ) and FGF15, mimicking therapeutic benefits of intestinal FXR inhibition. Interestingly, substitution with ruthenium­(II) in the analogue complex restored agonistic activity, highlighting metal-specific pharmacology. This iridium­(III)-driven platform integrates real-time FXR imaging with antagonism, offering a novel theranostic strategy for metabolic disorders such as NAFLD and IBD, and illuminates the potential of metallodrugs to modulate nuclear receptor function through precise metal–ligand coordination.

## 4 Conclusion and Outlook

Abnormal gene expression is related to diverse diseases, highlighting gene regulators as critical therapeutic targets. Over the past few decades, transition metal complexes, particularly octahedral iridium­(III) and rhodium­(III) organometallic complexes, have offered unique advantages in disrupting gene expression patterns due to their diverse structures, tunable photochemical properties, and capacity for specific biomolecular interactions. Their precisely defined steric scaffolds enable selective engagement with essential biomolecules, such as DNA and proteins, making them highly attractive candidates for developing organometallic modulators for gene regulation. Over the past decade, our group has systematically screened and developed a series of iridium­(III) and rhodium­(III) complexes from an extensive ligand library. These complexes directly modulate disease-associated gene expression and elucidate complex–biomolecule interactions, which are vital for spatiotemporal modulation. From disrupting STAT3 dimerization to engaging the VHL binding pocket, we tailored ligands and metal centers to fit distinct interfaces of gene regulators by modulating the polarity, steric hindrance, electrostatic interactions, and other parameters. However, synthesizing complexes from broad ligand libraries remains cumbersome due to the spatiotemporal distribution of gene regulators in cells and the diversity of their innate structures and interactions. Consequently, our recent efforts focused on developing metal-complex-based conjugates comprising three modular components: a metal complex scaffold, a variable linker, and a targeting unit. Conjugation often alters the endocytosis pathway and subcellular distribution of the targeting unit; for instance, the FXR conjugate inhibits nuclear translocation of FXR, converting the ligand from an agonist to an antagonist.^5^ In addition, leveraging the distinct membrane permeability and organelle-targeting properties of metal complexes, we designed a bifunctional lonidamine-conjugated organometallic iridium­(III) complex to target mitochondrial hexokinase 2 (HK2), facilitated by the mitochondria-targeting capability of the iridium­(III) scaffold.^66^ We anticipate that this indirect targeting strategy will enable more effective regulation of gene regulators through precise interventions.

Approved organic small-molecule drugs, like (+)-JQ-1 (binds to BET domain), vorinostat (binds to HDAC), and tazemetostat (binds to EZH2), typically exhibit high specificity for particular proteins like kinases or epigenetic regulators (IC_50_ as low as nanomolar).^67,68^ However, they generally bind to narrow pockets of active sites, making them less advantageous for disrupting PPIs. In contrast, octahedral iridium­(III) and rhodium­(III) complexes provide a larger interface capable of binding to essential domains of gene regulators, and they can even interact with unfolded proteins.^69^ Their conformation is governed by steric hindrance from van der Waals radii, resulting in a more flexible and less geometrically rigid structure. Additionally, small-molecule drugs typically require multistep synthesis and purification, while the organized three-dimensional architecture of coordination complexes can often be constructed via modular and convergent synthetic paths. Moreover, subcellular distribution also affects the activity of potential gene modulators. Small molecules primarily enter cells by passive diffusion according to their lipophilicity and p*K*_a_. This subcellular localization is often less directed and more diffuse throughout the whole cell unless specific targeting motifs are incorporated. Octahedral complexes are heavily influenced by their physicochemical properties (charge, lipophilicity, and ligand exchange kinetics). For example, hydrophobic and cationic complexes are often actively trafficked to mitochondria due to their negative mitochondrial membrane potential. Others may localize in the nucleus if they can cross the nuclear pore complex or in lysosomes due to endocytic uptake and sequestration. Through rational ligand design, the subcellular distribution of organometallic metal complexes can be tuned for organelle-specific targeting, while their unique optical properties provide intrinsic imaging capabilities. This combination of target modulation and biological tracking makes octahedral organometallic complexes, especially those based on iridium and rhodium, powerful platforms for modulating the activity and disrupting the PPIs of gene regulators.

Future developments of organometallic transition metal complexes for modulating gene regulators will rely on deeper insights into their dynamic interactions. Gene expression involves the assembly of numerous biomolecular hubs, necessitating the consideration of the specific spatiotemporal distribution of gene regulators within cells. The targeting unit enhances the metal conjugate affinity for gene regulators, which can be hijacked by metal-based organelle-targeting scaffolds. This relocates gene regulators away from their native cellular compartments, where PPI engagement or enzymatic reactions occur. Thus, organometallic conjugates interfere with gene expression by altering the subcellular distribution of the regulators. Furthermore, for interrupting and inactivating gene regulation processes, the organometallic moiety incorporates diverse C^∧^N and N^∧^N ligands. These impose steric resistance (e.g., spatial hindrance, electrostatic interference, and other non-covalent effects) between gene regulators and their substrates. Finally, we anticipate that leveraging biological processes and the rational design of organometallic conjugates will enable precise modulation of PPIs and biological activities of gene regulators, offering new perspectives for developing metal-based modulators.
